# Psychopathology is associated with reproductive health risk in European adolescents

**DOI:** 10.1186/s12978-018-0618-0

**Published:** 2018-11-06

**Authors:** Pietro Gambadauro, Vladimir Carli, Camilla Wasserman, Gergö Hadlaczky, Marco Sarchiapone, Alan Apter, Judit Balazs, Julio Bobes, Romuald Brunner, Doina Cosman, Christian Haring, Christina W Hoven, Miriam Iosue, Michael Kaess, Jean Pierre Kahn, Elaine McMahon, Vita Postuvan, Airi Värnik, Danuta Wasserman

**Affiliations:** 1 National Centre for Suicide Research and Prevention of Mental Ill-Health (NASP), Department of Learning, Informatics, Management and Ethics (LIME), Karolinska Institutet, 171 77 Stockholm, Sweden; 2 Department of Women’s and Children’s Health, Uppsala University, 75185 Uppsala, Sweden; 3 Res Medica Sweden, Gynaecology and Reproductive Medicine, 75224 Uppsala, Sweden; 4 Department of Child and Adolescent Psychiatry, Columbia University-New York State Psychiatric Institute, New York, USA; 5 Department of Medicine and Health Science, University of Molise, 86100 Campobasso, Italy; 6 National Institute of Health for Migration and Poverty, Via di San Gallicano 25/a, 00100 Rome, Italy; 7 Schneider Children’s Medical Center of Israel, Tel Aviv University, Tel Aviv, Israel; 8 Vadaskert Child Psychiatric Hospital and Outpatient Clinic, Budapest, 1021 Hungary; 9 Institute of Psychology, Eötvös Loránd University, Izabella u. 46, Budapest, 1064 Hungary; 10 Department of Psychiatry, University of Oviedo, CIBERSAM School of Medicine, Julian Claveria 6 - 3°, 33006 Oviedo, Spain; 11 Department of Child & Adolescent Psychiatry, Center for Psychosocial Medicine, University of Heidelberg, Blumenstrasse 8, D-69115 Heidelberg, Germany; 12 Clinical Psychology Department, Iuliu Hatieganu University of Medicine and Pharmacy, Cluj-Napoca, Romania; 13 Department for Psychiatry and Psychotherapy B, State Hospital Hall in Tyrol, Tirol-kliniken, Milser Straße 10, A- 6060 Hall, Austria; 14 Department of Epidemiology, Mailman School of Public Health, Columbia University, New York, USA; 15 Department of Psychiatry and Clinical Psychology, CHRU de NANCY and Pôle 6, Centre Psychothérapique de Nancy-Laxou, Université de Lorraine, NANCY, France; 16 National Suicide Research Foundation, University College Cork, Cork, Ireland; 17 Slovene Center for Suicide Research, Andrej Marusic Institute, University of Primorska, Muzejski trg 2, 6000 Koper, Slovenia; 18 Estonian-Swedish Mental Health & Suicidology Institute, Ctr. Behav. & Hlth. Sci, Tallinn University, Tallinn, Estonia

**Keywords:** Adolescence, Behaviour, Mental health, Psychopathology, Reproductive health, Sexual initiation, SEYLE study

## Abstract

**Background:**

Reproductive and mental health are key domains of adolescent wellbeing but possible interrelationships are poorly understood. This cross-sectional study evaluated the association between psychopathology and reproductive health risk among European adolescents.

**Methods:**

A structured self-report questionnaire was delivered to 12,395 pupils of 179 randomly selected schools in 11 European countries within the EU funded “Saving and Empowering Young Lives in Europe” (SEYLE) project. The questionnaire included items about sexual initiation and reproductive health risk factors, such as number of sexual partners, frequency of condom use, and pregnancy involvement. Psychopathology was evaluated with validated instruments and/or ad-hoc questions.

**Results:**

Of 11,406 respondents (median age 15; interquartile range [IQR] 14–15; 57% females), 18.8% reported sexual initiation. Sixty percent of them also reported at least one reproductive risk factor. Sexual initiation was significantly more common among pupils older than 15 years (38% versus 13.2% younger pupils) and males (21.3% versus 16.9% females). It was also more common among pupils with depression (age/sex-adjusted odds ratio [aOR] 1.871), anxiety (aOR 2.190), severe suicidal ideation (aOR 2.259), self-injurious behaviour (aOR 2.892), and suicide attempts (aOR 3.091). These associations were particularly strong among pupils ≤15 years old and, for overt psychopathology, among pupils with low non-sexual risk behaviour profile and females. Depression (aOR 1.937), anxiety (aOR 2.282), severe suicidal ideation (aOR 2.354), self-injurious behaviour (aOR 3.022), and suicide attempts (aOR 3.284) were associated with higher reproductive health risk, defined by an increasing number of coexisting reproductive risk factors.

**Conclusions:**

These findings suggest an alignment between mental and reproductive health risk and support the value of cross-domain collaboration in adolescent health. The association between psychopathology and reproductive health risk, as well as its variations with age, sex, and associated risk behaviours, should be considered when designing health-promoting or disease-preventing interventions for adolescents.

## Abstract in Spanish

**Antecedentes:**

La salud reproductiva y la salud mental son dominios clave del bienestar de los adolescentes, pero las posibles interrelaciones son poco conocidas. Este estudio transversal evaluó la asociación entre la psicopatología y el riesgo para la salud reproductiva entre los adolescentes europeos.

**Métodos:**

Se entregó un cuestionario estructurado de autoinforme a 12.395 alumnos de 179 escuelas seleccionadas al azar en 11 países europeos dentro del proyecto “Saving and Empowering Young Lives in Europe” (SEYLE), financiado con fondos comunitarios de la Unión Europea. El cuestionario incluyó preguntas sobre la iniciación sexual y los factores de riesgo para la salud reproductiva, como el número de parejas sexuales, la frecuencia del uso del condón y el haber estado involucrado en embarazos. La psicopatología se evaluó con instrumentos validados y/o preguntas ad-hoc.

**Resultados:**

De 11.406 encuestados (mediana de edad 15, rango intercuartílico [IQR] 14–15, 57% mujeres), 18.8% informaron iniciación sexual. Sesenta por ciento de ellos también informaron al menos un factor de riesgo reproductivo. La iniciación sexual fue significativamente más común entre los alumnos mayores de 15 años (38% versus 13.2% en alumnos más jóvenes) y los varones (21.3% versus 16.9% en mujeres). También fue más común entre los alumnos con depresión (odds ratio ajustado por edad/sexo [aOR] 1.871), ansiedad (aOR 2.190), ideación suicida grave (aOR 2.259), comportamiento autolesivo (aOR 2.892) e intentos de suicidio (aOR 3.091). Estas asociaciones resultaron particularmente fuertes entre los alumnos de ≤15 años de edad y, para la psicopatología manifiesta, entre los alumnos sin comportamiento de riesgo no sexual y las mujeres. La depresión (aOR 1.937), la ansiedad (aOR 2.282), la ideación suicida severa (aOR 2.354), el comportamiento autolesivo (aOR 3.022) y los intentos de suicidio (aOR 3.284) se asociaron con un mayor riesgo para la salud reproductiva, definido por un número creciente de factores de riesgo reproductivo coexistentes.

**Conclusiones:**

Estos hallazgos sugieren una alineación entre los riesgos para la salud mental y reproductiva, y respaldan el valor de la colaboración multidisciplinar en la salud de los adolescentes. La asociación entre la psicopatología y el riesgo para la salud reproductiva, así como sus variaciones con la edad, el sexo y los comportamientos de riesgo asociados, deben considerarse al diseñar intervenciones de promoción de la salud o prevención de enfermedades para adolescentes.

## Abstract in French

**Contexte:**

La santé mentale et de la reproduction sont des domaines clé du bien-être des adolescents mais les possibles interactions entre ces deux domaines sont encore mal connues. Cette étude transversale a évalué l’association entre la psychopathologie et les risques de santé de la reproduction chez des adolescents européens.

**Méthodes:**

Un auto-questionnaire structuré a été soumis à 12.395 élèves de 179 écoles recrutées de façon aléatoire, dans 11 pays européens au cours d’une étude financée dans le cadre du 7° appel d’offre européen (FP7), l’étude SEYLE (« *Saving and Empowering Young Lives in Europe* »). Le questionnaire comprenait des questions relatives à l’initiation de la vie sexuelle et aux facteurs de risque liés à la santé de la reproduction, tels que le nombre de partenaires sexuels, la fréquence de l’utilisation de préservatifs, la survenue de grossesses. Les données psychopathologiques ont été évaluées à l’aide d’instruments validés et/ou de questions spécifiques.

**Résultats:**

Parmi les 11.406 élèves ayant répondu ( âge moyen 15 ans, gamme interquartile ▪IQR▪ 14-15 ; 57% de femmes), 18,8% avaient initié une vie sexuelle. Soixante pour cent d’entre eux mentionnaient au moins un facteur de risque reproductif. L’initiation sexuelle était significativement plus fréquente parmi les élèves âgés de plus de 15 ans (38% vs 13,2%, chez les plus jeunes), chez les garçons (21,3% vs 16,9% chez les filles). Elle était également plus fréquente parmi les élèves présentant des symptômes de dépression (odds ratios ajustés pour l’âge et le sexe ▪aOR▪ 1,871), d’anxiété (aOR : 2,190), des idées suicidaires sévères (aOR : 3,091), des conduites d’automutilation (aOR : 2,892) et des tentatives de suicide (aOR : 3,091). Ces associations étaient particulièrement fortes parmi les élèves âgés de 15 ans ou moins et, pour la psychopathologie avérée, parmi les élèves présentant des profils de faibles comportements à risque non sexuels et les filles. La dépression (aOR : 1,937), l’anxiété (aOR : 2,282), les idées suicidaires sévères (aOR : 2,354), les conduites d’automutilation (aOR : 3 ?022) et les tentatives de suicide (aOR : 3,284) étaient associés à un risque de santé reproductive plus élevé, défini par un nombre croissant de facteurs de risque co-existants.

**Conclusions:**

Ces résultats suggèrent un parallèle entre les risques de santé mentale et de la reproduction et témoignent de l’intérêt de collaborations interdisciplinaires en matière de santé des adolescents. L’association entre les facteurs de risque de santé mentale et de la reproduction ainsi que leurs variations avec l’âge, le sexe et les facteurs de risques associés devraient être pris en compte dans l’élaboration d’interventions de promotion de la santé et de prévention des maladies, chez les adolescents.

## Abstract in German

**Hintergrund:**

Reproduktive und psychische Gesundheit sind Schlüsselbereiche des jugendlichen Wohlbefindens, aber mögliche Zusammenhänge sind kaum verstanden. Diese Querschnittsstudie untersuchte den Zusammenhang zwischen Psychopathologie und reproduktivem Gesundheitsrisiko bei europäischen Jugendlichen. Methoden: Im Rahmen des von der EU-Kommission geförderten Projektes "Saving and Empowering Young Lives in Europe" (SEYLE) wurden 12.395 Schüler von 179 zufällig ausgewählten Schulen in 11 europäischen Ländern mit einem strukturierten Fragebogen befragt. Der Fragebogen umfasste Fragen zur sexuellen Initiation und Risikofaktoren der reproduktiven Gesundheit, wie z. B. die Anzahl der Sexualpartner, Häufigkeit der Kondomnutzung und Schwangerschaften. Die psychopathologischen Auffälligkeiten wurden mit validierten Instrumenten und / oder Ad-hoc-Fragen evaluiert.

**Ergebnisse:**

Von 11.406 Befragten (Median 15; Interquartilbereich [IQR] 14-15; 57% Mädchen) gaben 18,8% an sexuell aktiv geworden zu sein. Sechzig Prozent von ihnen berichteten auch mindestens einen reproduktiven Risikofaktor. Sexuelle Initiation war signifikant häufiger bei Schülern, die älter als 15 Jahre waren (38% versus 13,2% jüngeren Schülern) und bei Jungen (21,3% versus 16,9% Mädchen). Eine sexuelle Initiation lag auch häufiger bei Schülern mit Depressionen (Alter / Geschlechts-adjustiertes Odds Ratio [aOR] 1.871), Angstsymptomen (aOR 2.190), schweren Suizidgedanken (aOR 2.259), selbstverletzendem Verhalten (aOR 2.892) und Selbstmordversuchen (aOR 3.091) vor. Diese Assoziationen waren besonders ausgeprägt bei Schülern ≤15 Jahren und bei deutlich auffälliger Psychopathologie bei Schülern mit geringem nicht-sexuellem Risikoverhalten und beim weiblichen Geschlecht. Depressive Symptome (aOR 1.937), Angstsymptome (aOR 2.282), schwere Suizidgedanken (aOR 2.354), selbstverletzendes Verhalten (aOR 3.022) und Suizidversuche (aOR 3.284) waren mit einem erhöhten Risiko für die reproduktive Gesundheit verbunden - definiert durch eine steigende Anzahl von koexistierenden reproduktiven Risikofaktoren.

**Schlussfolgerungen:**

Diese Ergebnisse deuten auf eine Beziehung zwischen Risiken für die psychische und reproduktive Gesundheit hin und unterstützen den Wert der domänenübergreifenden Zusammenarbeit bei der Gesundheit von Jugendlichen. Der Zusammenhang zwischen Psychopathologie und reproduktivem Gesundheitsrisiko sowie seine alters- und geschlechtsspezifischen Unterschiede und damit verbundenem Risikoverhalten sollten bei der Gestaltung gesundheitsfördernder oder krankheitsvorbeugender Maßnahmen für Jugendliche berücksichtigt werden.

## Plain English summary

Reproductive and mental health are key domains of adolescent wellbeing but possible interrelationships are poorly understood. This study evaluated the association between psychopathology and reproductive health risk in a large multinational sample of European adolescents. A structured self-report questionnaire was delivered to 12,395 pupils of 179 randomly selected schools in 11 European countries within the EU funded “Saving and Empowering Young Lives in Europe” (SEYLE) project. Among 11,406 respondents, a wide spectrum of psychopathologic manifestations was positively associated with sexual initiation, independently of age and sex. The association appeared to be stronger for more overt manifestations, such as self-injurious behaviour and suicide attempts. However, having depression or anxiety was also associated with sexual initiation, consistently with a dimensional nature of adolescent psychopathology. The association between overt psychopathology and sexual initiation was stronger among pupils with low non-sexual risk behaviours compared to those with high non-sexual risk behaviours. Additionally, there was a stronger association between depression, serious suicidal ideation, self-injurious behaviour, or suicide attempts, and sexual initiation among pupils ≤15 years old compared to their older counterparts. Similarly, the association between anxiety or self-injurious behaviour and sexual initiation was stronger among girls compared to boys. Finally, pupils with psychopathology manifestations were more likely to have an increased reproductive health risk.

These findings suggest an alignment between mental and reproductive health risk and support the value of cross-domain collaborative efforts to prevent disease and improve the health of young people.

## Background

Adolescence is a unique period of human life characterized by dramatic development, which includes reproduction-related changes and, often, sexual initiation. Developing sexuality is physiologic during adolescence, but early debut implies a risk for immediate or future adverse reproductive health outcomes, such as unwanted teenage pregnancies and their consequences [1, 2]. Unsafe sexual practices expose to the risk of sexually transmitted infections, which can also have repercussions on future health and fertility [3–6]. Women who had sexual debut before the age of 15, more often report gaps in contraceptive use, sex-partner concurrency, and serial monogamy later in life [4, 7, 8]. Apart from reproductive health risk outcomes, early sexual initiation is associated with non-sexual risk behaviours (e.g. smoking or substance abuse), context vulnerabilities (e.g. bullying, truancy and low parental involvement) and psychological difficulties [9], which are not uncommon among girls and boys [10].

While reproductive and mental health are acknowledged key aspects of European adolescents’ current and future wellbeing [11], both appear to be neglected in terms of systematic data collection and research in Europe. Many studies focus on health outcomes belonging to one specific domain (e.g. reproductive or mental), but likely interrelationships between different aspects of adolescent health are still poorly studied hence overlooked by ad-hoc policies [12]. However, the potential outcomes of adolescent sexual behaviours are obviously not only physical but also mental and social [13]. An association between early sexual initiation and psychopathology manifestations such as depression has been reported by studies that mostly proceed from the USA [5, 14–17], whereas fewer similar findings are available from European countries [18, 19]. Although there is evidence that early sexual debut may lead to adverse mental health outcomes [17], depression has also been observed to precede and predict sexual initiation, multiple partners and inconsistent condom use among adolescents [5, 16, 18]. Other longitudinal studies have found that mental health problems predict poor compliance to prescribed contraception [20] and depressive symptoms are associated with an increased risk of unintended pregnancies [21].

The complex and likely bidirectional relationship between mental health and sexual behaviours is consistent with the conceptual view that factors influencing sexual activity also determine the way adolescents perceive and evaluate their own behaviour [13]. While sexual behaviours may directly lead to physical health outcomes, it is their perception that arguably mediates outcomes in the mental and social health domains [13]. In view of this complexity, a broader perspective on adolescents’ sexual experiences taking into account other facets of their wellbeing, such as mental health, would be desirable in Europe. It would also be important to study whether other psychopathology manifestations beyond depression are associated with early sexual initiation and risk-taking, although that would clearly require more substantial research efforts. This study took advantage of comprehensive information about the health and wellbeing of more than 12,000 European adolescents, which was gathered by a recent EU-funded project called SEYLE (Saving and Empowering Young Lives in Europe) [22, 23]. We aimed at evaluating whether pupils with psychopathology manifestations, such as depression, anxiety, self-injurious behaviour and suicidality, are more likely to have a higher reproductive health risk, defined by earlier sexual initiation and associated risk factors. A secondary objective was to verify whether the strength of the association between psychopathology and sexual initiation is modified by age, sex or the co-occurrence of non-sexual risk behaviours.

## Methods

### Study population

Between 2009 and 2011, 12,395 adolescents (median age 15; interquartile range [IQR] 14–15; mean age 14.91 ± 0.90) were enrolled into the EU-funded SEYLE project [22]. This population consisted of young females (55.2%) and males (44.8%) attending 179 schools in 11 countries: Austria, Estonia, France, Germany, Hungary, Ireland, Israel, Italy, Romania, Slovenia and Spain. Karolinska Institutet, Sweden, acted as scientific coordinating centre, and the Child Psychiatric Epidemiology Group at Columbia University and New York State Psychiatric Institute participated as methodology experts. The two core objectives of SEYLE were to gather epidemiological data about European adolescent health and wellbeing, and to actively test suicide-preventive interventions in a randomized controlled trial [24]. The included schools were randomly selected out of a list of eligible schools located within the study sites of each country, according to the selection criteria described by Wasserman et al. [22]. A 67.8% response rate was obtained from the selected schools. Elsewhere published analyses show that SEYLE’s study sites were representative of each respective national population, based on none or small significant differences with the respective country in several key parameters, including age, sex distribution, proportion of 15-year old males and females, net income, immigrants and unemployment rates [23]. At recruitment, pupils were asked to complete a structured, self-report questionnaire addressing socio-demographics, risk factors, lifestyle, and mental health. This paper-based survey was administered in the official national languages during a single classroom session. Research staff was available in each location to supervise and assist the pupils with the process. For the current study, we selected all SEYLE cases with complete baseline data about age, sex, and reproductive health risk (N 11,406; 92.5%).

The consent of pupils and their caregivers was obtained before inclusion. Further information about the sample and SEYLE’s core methodology is elsewhere published [22, 23].

### Outcome variable

The main outcome in the study was the prevalence of self-reported sexual initiation, which was evaluated by one closed-ended question (“have you ever had sexual intercourse?”). SEYLE’s questionnaire investigated reproductive risk factors (RRF), that were included in analysis as dichotomous variables: number of sexual partners (dichotomized as 1 versus ≥2); use of condom (dichotomized as “rarely or never” versus “always or almost all the time”); pregnancy involvement (yes or no). For each study subject, an additional outcome variable called “reproductive health risk” was computed by subcategorizing the outcome sexual initiation depending on associated risk factors (no initiation; initiation without RRF; initiation and 1 RRF; initiation and 2 or 3 RRF).

### Psychopathology measurements

Psychopathology was evaluated with known instruments such as the Zung Self-Rating Anxiety Scale (Z-SAS) [25], the modified 20-item Beck Depression Inventory-II (BDI-II) [26–28], a modified six-item version of the Deliberate Self-Harm Inventory (DSHI) [29, 30], and the Paykel Suicide Scale (PSS) [31]. When official validated versions were unavailable in the required language, the surveys were translated, back-translated, and linguistically adapted. Internal reliability of psychometric scales was confirmed by a consistently high Cronbach’s alpha [23]. Dichotomous psychopathology variables were computed for each study subject. Anxiety and depression were respectively defined by a Z-SAS score ≥ 45 and a modified BDI-II score ≥ 17. Self-injurious behaviour was defined by a sum of ≥3 obtained adding all points of the modified DSHI [10]. Severe suicide ideation was defined as having seriously considered taking one’s own life, or having made plans about that, at least sometimes during the previous two weeks (4th item of the PSS) [24]. Self-reported suicide attempts were investigated by means of a multiple choice closed-ended question (“Have you ever tried to take your own life?”; possible answers: yes, during the past 2 weeks; yes, during the past 6 months or longer; no, never) and recoded as a dichotomous variable (yes or no).

### Potential confounders and effect modifiers

Self-reported age and sex of each participant were obtained through SEYLE’s questionnaire. Age was dichotomized with a 15-year-old cut-off (≤15 versus > 15), based on evidence that sexual debut in Europe more often occurs after 15 years old [32]. Besides, other large surveys collect data on sexual initiation by the age of 15 and European teenagers believe that one may be “too young to have sexual intercourse” at an average age of 15.5 [33].

SEYLE’s questionnaire also provided data regarding non-sexual risk behaviours such as substance abuse (smoking, alcohol and illegal drugs), truancy, sedentariness, poor sleep, and high media use. Previous latent class analysis research identified different patterns of risk clustering in the SEYLE cohort [10]. Building up on those findings, participants in the present study were sub-grouped according to their non-sexual risk behaviour profile into a low risk profile group, including pupils with low scores on all examined risk behaviours; and a high risk profile group, including pupils clustering on multiple overt (i.e. smoking, alcohol and illegal drugs use, truancy) and/or “invisible” (i.e. poor sleep, sedentariness and high media use) risk behaviours [10].

### Statistical analyses

Descriptive statistics were used to describe the study population, with a focus on the prevalence of reported sexual initiation, reproductive risk factors, and psychopathology. Chi-square test was used to compare the prevalence of reported sexual initiation depending on age, sex, non-sexual risk behaviour profile, and psychopathological manifestations, as previously defined. The association between each psychopathology variable and sexual initiation was tested with multivariable logistic regression analyses. These analyses were repeated after stratifying the sample for age and sex, as well as for the two subgroups of opposite non-sexual risk behaviour profile. Additional analyses including interaction terms were performed when the stratified findings suggested differences among the strata, in order to test interactions between each psychopathology manifestation and the variables age, sex or non-sexual risk behaviour profile. Ordinal and multinomial logistic regression analyses were used to measure the strength of association between psychopathology variables and the outcome reproductive health risk. The logistic regression analyses were by default adjusted for the age and sex variables because of the expected association with the study’s main outcome measure, with the exception of the stratified analyses where adjustment was not applicable. Statistical significance of differences was set at a two-tailed *p* value of less than 0.05. Odds ratios (OR) were calculated together with 95% confidence intervals (CI). The software IBM® SPSS® Statistics ver. 23 for macOS was used for statistical analyses.

### Ethical approval

SEYLE was approved by the European Commission, as a precondition for funding, as well as by the local research ethics committees of each national recruiting centre [22]. An external advisor from the University of Basel, Switzerland, provided independent ethical assessment and supervision of the project [23].

### Clinical trial registration

SEYLE was registered in the German Clinical Trials Register (DRKS00000214).

## Results

Complete data from SEYLE’s reproductive health items were available from 11,406 pupils, accounting for the 92.5% of the whole SEYLE population (median age 15; IQR 14–15; mean age 14.88 ± 0.88). Forty-three percent (N 4889) of the respondents were males, while 57% (N 6517) were females (Table 1). The majority of respondents were 15 years old or younger (N 8844; 77.2%).

**Table 1:** Age, sex and psychopathology in 11,406 adolescents with or without sexual initiation

Variables	Categories	N of respondents^a^	Sexual initiation		
			N Yes (%)	N No (%)	*p*-value^b^
Age	≤15 > 15	8844 2562	1169 (13.2) 974 (38)	7675 (86.8) 1588 (62)	< 0.001
Sex	Females Males	6517 4889	1101 (16.9) 1042 (21.3)	5416 (83.1) 3847 (78.7)	< 0.001
Non-sexual risk behaviours profile	high low	2692 5529	556 (20.7) 1008 (18.2)	2136 (79.3) 4521 (81.8)	0.009
Anxiety	yes no	820 10,274	257 (31.3) 1858 (18.1)	563 (68.7) 8416 (81.9)	< 0.001
Depression	yes no	1308 9983	370 (28.3) 1750 (17.5)	938 (71.7) 8233 (82.5)	< 0.001
Self-injurious behaviour	yes no	1012 10,243	369 (36.5) 1740 (17)	643 (63.5) 8503 (83)	< 0.001
Severe suicidal ideation	yes no	426 10,893	142 (33.3) 1984 (18.2)	284 (66.7) 8909 (81.8)	< 0.001
Suicide attempts	yes no	430 10,875	172 (40) 1948 (17.9)	258 (60) 8927 (82.1)	< 0.001

*See text for definitions of psychopathology variables*

^*a*^ *Response rates (N/11,406) are as follows: age 100%; sex 100%; non-sexual risk behaviours 72.1%; anxiety 97.3%; depression 99%; self-injurious behaviour 98.7%; severe suicidal ideation 99.2%; suicide attempts 99.1%*

^*b*^ *P-values were assessed by chi-square tests*

### Sexual initiation

Sexual initiation was reported by 18.8% of the respondents (N 2143; mean age 15.39 ± 0.87). It was significantly more common among boys (21.3% versus 16.9% in girls; *p* < 0.001) as well as among those older than 15 years old (38.0% versus 13.2% in pupils ≤15 years old; *p* < 0.001). The rate of sexual initiation was also significantly higher in the high non-sexual risk behaviours profile group (20.7% versus 18.2% in the low profile group; *p* = 0.009).

**Fig. 1:**
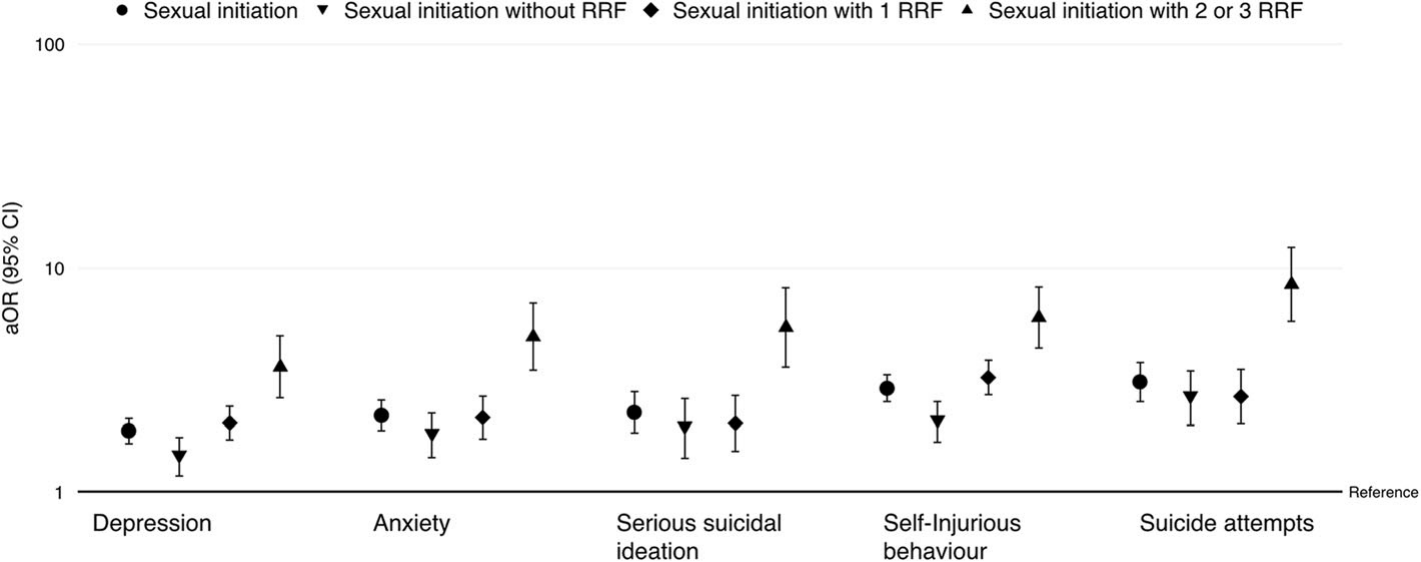
Psychopathology and sexual initiation among European adolescents. Logistic regression derived odds ratios (aOR) and 95% confidence of interval (95% CI) of reported sexual initiation, with or without associated reproductive risk factors (RRF) in European adolescents with different manifestations of psychopathology. The reference category of the outcome is “no sexual initiation”. The odds ratios are adjusted for age and sex

More than 97% of participants responded to each one of the considered mental health items (Table 1). The prevalence of reported sexual initiation was significantly higher among pupils with anxiety, depression, self-injurious behaviour, severe suicidal ideation and suicide attempts, as defined in the previous section (Table 1). All psychopathology manifestations were significantly associated with reported sexual initiation even when adjusting for age and sex (Table 2; Fig. 1). The results of the logistic regression analyses remained unaltered even when adjusting for pupils’ country of origin. Stratified analyses suggested a stronger association among pupils with a low non-sexual risk behaviour profile (Table 2). Further analyses with interaction terms confirmed a significantly stronger association between self-injurious behaviour and sexual initiation among pupils with low non-sexual risk behaviours compared to those with high non-sexual risk behaviours (*p* = 0.024). Similarly, the association between suicide attempts and sexual initiation in the low non-sexual risk behaviours group was significantly stronger than in the high non-sexual risk behaviours group (*p* = 0.038).

**Table 2:** Prevalence and odds of sexual initiation among European adolescents with and without psychopathology

		Total sample		Low non-sexual risk behaviours profile^b^		High non-sexual risk behaviours profile^c^	
		%	OR (95% CI)^a^	%	OR (95% CI)^a^	%	OR (95% CI)^a^
Depression	yes no	28.3 17.5	1.871 (1.628–2.150)	28.6 16.9	2.004 (1.642–2.446)	30.5 19.4	1.792 (1.360–2.362)
Anxiety	yes no	31.3 18.1	2.190 (1.856–2.586)	30.1 17.6	2.211 (1.732–2.821)	32.0 19.8	1.893 (1.360–2.633)
Severe suicidal ideation	yes no	33.3 18.2	2.259 (1.816–2.809)	34.0 17.7	2.434 (1.767–3.352)	33.6 19.9	1.972 (1.293–3.007)^d^
Self-injurious behaviour	yes no	36.5 17.0	2.892 (2.500–3.345)	37.6 16.4	3.361 (2.732–4.134)	34.1 19.1	2.177 (1.622–2.921)
Suicide attempts	yes no	40.0 17.9	3.091 (2.504–3.816)	41.9 17.4	3.840 (2.824–5.223)	36.8 19.9	2.196 (1.430–3.371)

^a^Logistic regression derived odds ratios (OR) and 95% confidence intervals (CI) measuring the strength of association between each psychopathological variable and the outcome “sexual initiation”. Odds ratios are adjusted for age and sex. All *p*-values are < 0.001 except for ^d^ *p* = 0.002

^b^Pupils scoring low on all non-sexual risk behaviours

^c^Pupils clustering on multiple non-sexual risk behaviours

The age-sex stratified analysis showed particularly strong associations between psychopathology and sexual initiation among younger (versus older) and female (versus male) pupils (Table 3). Therefore, we performed separate analyses including interaction terms which confirmed a significantly stronger (*p* < 0.05) association between depression, serious suicidal ideation, self-injurious behaviour, or suicide attempts, and sexual initiation among pupils ≤15 years old compared to their older counterparts. Similarly, the association between anxiety or self-injurious behaviour and sexual initiation was significantly stronger (*p* < 0.05) among girls compared to boys.

**Table 3:** Association between psychopathology and sexual initiation, stratified for age and sex

Independent variable	Age	Females OR (95% CI)^a^	Males OR (95% CI)^a^	Both sexes OR (95% CI)^a, b^
Depression	≤15 years > 15 years All ages ^c^	2.235 (1.824–2.740)*** 1.542 (1.167–2.038)** 1.960 (1.660–2.315)***	2.267 (1.652–3.112)*** 1.118 (0.760–1.644)° 1.686 (1.309–2.171)***	2.245 (1.891–2.664)*** 1.380 (1.101–1.730)**
Anxiety	≤15 years > 15 years All ages ^c^	2.702 (2.150–3.397)*** 2.091 (1.519–2.880)*** 2.476 (2.052–2.989)***	1.644 (1.051–2.572)* 1.216 (0.695–2.125)° 1.458 (1.022–2.080)*	2.416 (1.973–2.960)*** 1.829 (1.385–2.414)***
Severe suicidal ideation	≤15 years > 15 years All ages ^c^	3.156 (2.305–4.322)*** 1.316 (0.825–2.100)° 2.369 (1.807–3.105)***	2.568 (1.655–3.986)*** 1.386 (0.753–2.550)° 2.070 (1.433–2.992)***	2.939 (2.275–3.796)*** 1.341 (0.926–1.943)°
Self-injurious behaviour	≤15 years > 15 years All ages ^c^	3.723 (2.993–4.630)*** 2.595 (1.873–3.594)*** 3.330 (2.770–4.002)***	2.753 (2.070–3.661)*** 1.618 (1.084–2.415)* 2.298 (1.811–2.916)***	3.321 (2.793–3.948)*** 2.154 (1.672–2.774)***
Suicide attempts	≤15 years > 15 years All ages ^c^	4.624 (3.460–6.179)*** 1.900 (1.253–2.881)** 3.449 (2.693–4.417)***	3.422 (2.128–5.504)*** 1.184 (0.618–2.269)° 2.334 (1.562–3.488)***	4.253 (3.319–5.449)*** 1.654 (1.165–2.349)**

*p*-values ° ≥0.05; * < 0.05; ** < 0.01; *** < 0.001

^a^Logistic regression derived odds ratios (OR) and 95% confidence intervals (CI). ^b^ Adjusted for sex. ^c^ Adjusted for age

### Reproductive health risk

Sexual experience was associated with at least one of the considered reproductive risk factors in 60% of the cases: 52.4% reported more than one sexual partner (40.1% of females and 65.4% of males); 14.7% reported rare/no use condoms (18.3% of females and 10.8% of males); and 3% reported pregnancy involvement (2.5% of females and 3.6% of males) (Table 4). Half of the adolescents reported one reproductive risk factor, while 9% reported two, and 0.6% reported all the three considered risk factors (Table 4).

**Table 4:** Characteristics of 2143 adolescents reporting sexual initiation and associated risk factors

	N	%
Sexual initiation	2143	100
Sex		
Males	1042	48.6
Females	1101	51.4
Age		
≤ 15	1169	54.5
> 15	974	45.5
Reproductive risk factors		
More than 1 partner	1123	52.4
Rare or no condom use	314	14.7
Pregnancy	65	3.0
Cases according to number of reproductive risk factors (RRF)		
No RRF	858	40.0
1 RRF	1080	50.4
2 or 3 RRF	205	9.6

Having each psychopathology manifestation significantly increased the odds of being in higher levels of the outcome reproductive health risk (Table 5). This was particularly evident when the outcome was sexual initiation with multiple RRF (versus no sexual initiation) in the multinomial analyses (Fig. 1).

**Table 5:** Association between psychopathology and reproductive health risk

	Estimates	Odds ratios	95% confidence interval	
			Lower	Upper
Depression	0.661	1.937	1.692	2.217
Anxiety	0.825	2.282	1.944	2.678
Severe suicidal ideation	0.856	2.354	1.910	2.901
Self-injurious behaviour	1.106	3.022	2.630	3.476
Suicide attempts	1.189	3.284	2.691	4.007

Ordinal logistic regression derived estimates, odds ratios and 95% confidence intervals, adjusted for age and sex

All *p* values are < 0.001

## Discussion

This study evaluated the association between psychopathology and reproductive health risk in a large, multinational and representative sample of more than 11,000 European adolescents. A wide spectrum of psychopathologic manifestations was positively associated with sexual initiation, independently of age and sex. The association appeared to be stronger for more overt manifestations, such as self-injurious behaviour and suicide attempts. However, having depression or anxiety was also associated with sexual initiation, consistently with a dimensional nature of adolescent psychopathology [28]. The association between overt psychopathology and sexual initiation was stronger among pupils with low non-sexual risk behaviours compared to those with high non-sexual risk behaviours. Additionally, there was a stronger association between depression, serious suicidal ideation, self-injurious behaviour, or suicide attempts, and sexual initiation among pupils ≤15 years old compared to their older counterparts. Similarly, the association between anxiety or self-injurious behaviour and sexual initiation was stronger among girls compared to boys. Finally, pupils with psychopathology manifestations were more likely to have an increased reproductive health risk.

Physiological reproductive and sexual development characterizes the adolescent transition from childhood to adulthood. Females and males become fertile during this period when many have also their sexual initiation. In European countries, sexual debut often occurs between 16 and 18 years of age, while it is less common before the age of 15 [32]. This was confirmed by our findings since only 13% of adolescents ≤15 years old reported sexual debut compared to 38% of those over 15 years old of age. The evident link between sexual behaviours and the risk of adverse reproductive health outcomes is often targeted by research and policy efforts in the field of adolescent health. The idea of early sexual debut as a gateway to sexual and reproductive risk has commonly informed responses directed to avoiding or postponing sexual activity, or to minimizing risks through specific education and care. However, the timing of sexual initiation alone may not be considered as an independent marker of risk, as risky sexual activity is often associated with non-sexual risk behaviours (e.g. substance abuse) [3, 9, 34, 35], exposure to childhood trauma [9, 34, 36] or problematic social interactions (e.g. with peers, school or family) [9, 34]. Therefore, a reductionist perspective of reproductive health as an isolated domain may only offer a limited understanding of adolescent risk behaviour and is hardly functional to the development of much needed and advocated intersectoral and multicomponent actions for adolescent wellbeing [37].

Aspects of the relationship between sexual and non-sexual risk behaviours may be interpreted through a gateway effect (e.g. substance abuse or victimization facilitating exposure to unsafe sex) [12]. However, such interpretation overlooks the fact that adolescents often cluster on multiple risk behaviours, which leads to ineffective surveillance and responses [12]. An alternative or complementary interpretation may be informed by a behavioural risk syndrome model, which frames adolescent risk-taking in the context of broader correlates, such as individual and social vulnerabilities [12, 38–40]. Therefore, our initial hypothesis that adolescent psychopathology could be associated with early/risky sexual activity appeared to be well grounded. Nevertheless, most related observations proceed from the USA and focus on depression [5, 15–17, 41, 42], whereas less studies have considered young Europeans [18, 19] or other psychopathology manifestations such as suicidality [14, 19].

The present study adds insights to existing literature: first of all, the association between psychopathology and sexual initiation exists also among European adolescents, and involves a wider range of manifestations beyond depression; secondly, our findings suggest an alignment between the two domains of mental and reproductive health, as the strength of association increases with more overt psychopathologic manifestations or higher reproductive health risk. Another peculiarity of this study is that it considered factors that, by altering the strength of the association between psychopathology and sexual initiation, may inform our interpretation. The stronger association among those who report sexual debut by the age of 15 is arguably explained by the fact that sexual activity is less normative at younger age. It therefore occurs more often among vulnerable adolescents, who also engage in other risk behaviours and are exposed to worse health outcomes during adolescence and adult life. Similarly, the strong association in case of overt psychopathology observed among young females, who generally experience a later sexual debut [32, 43], suggests that norm-breaking behaviours may relate to dysfunctional situations where risk-taking and poorer mental health coexist.

Finally, the stronger association between overt psychopathology and sexual initiation among pupils with low risk behaviour profile is an interesting and partially unexpected finding. In the absence of psychopathology, the higher prevalence of sexual initiation among pupils engaging in multiple non-sexual risk behaviours may relate to gateway mechanisms (e.g. non-sexual behaviours as facilitators to risky sex) and is consistent with the known clustering of risk behaviours among undercontrolling adolescents with conduct and hyperactivity (i.e. externalizing) problems [10, 44]. On the contrary, sexual initiation was most commonly reported by pupils with psychopathology, independently of their non-sexual risk behaviour profile. Therefore, internalizing symptoms, such as the ones captured by this study, may play a specific role when it comes to sexual behaviours, and the possibility of a bidirectional relationship should be acknowledged. Altogether, these findings explain why targeting individual risk behaviours without addressing underlying vulnerabilities, such as poor mental health, may be an ineffective way to promote adolescent well-being.

The present study has some limitations which should be taken into account. Not all SEYLE participants could be included because of missing data regarding the reproductive health outcome measures. The amount of excluded participants was however very low, as 92% were eventually included. Additionally, the response rates to the psychopathology measurements were consistently very high (97–99%). In terms of generalizability, the samples recruited at the eleven study sites were population-based and fairly representative at national level [23], though it should be acknowledged that Europe is a wide and heterogeneous geopolitical area. It is however reassuring that the association between depression and sexual initiation has also been observed in countries not participating to SEYLE [18], and that the clustering and psychosocial correlates of adolescent risk behaviours appear to be consistent across European countries [40]. Regarding the study methodology, a cross-sectional design does not allow for inferences about directionality or causality. A further consideration needs to be made regarding the study variables, which were obtained through self-report. Diagnostic interviews would allow for a more correct classification of psychiatric disorders, but they may be unfeasible in the context of a large study as well as they may underestimate symptoms. Self-report of risk or sexual behaviours may be biased by social pressure, although SEYLE participants were made aware about the strictly confidential procedures and anonymous data management and analysis. Respondents may have variably interpreted what “sexual intercourse” is, as no distinction between different sexual activities was made in the survey. Besides, our choice of reproductive health risk variables was restricted to those available in SEYLE’s questionnaire, while it would have been interesting to collect additional data, such as about contraceptive use, sexual orientation, gender identity, or exposure to sex-related trauma. Finally, SEYLE did not collect individual data about the age of sexual debut nor about pubertal timing, which may be a correlate of adolescent sexual experiences.

Despite those considerations, a number of implications for further research and practice may be suggested by this large and multinational study. It would be interesting to test our findings in longitudinal studies, as insights about the directionality of the relationship between psychopathology and reproductive health risk are needed to verify how much policy responses may rely on gateway models [12]. However, establishing directionality does not prove causality nor it ensures that interventions on the presumed gateway would have downstream effects on the outcome. Besides, the possibility of reverse or bidirectional relationships should be considered.

As a matter of fact, psychological vulnerabilities are possibly one of the numerous determinants of adolescents’ sexual behaviours and their self-evaluation, which is then mediating mental and social health outcomes [13]. A comprehensive view on those factors would help us understanding why physiologic events such as sexual debut and developing sexuality sometimes deviate from being positive experiences, as they instead could and should be [13]. Instead of addressing adolescent sexual behaviours only in view of their potential negative outcomes on physical health, it should therefore be acknowledged that coexisting problems in different domains of adolescent wellbeing may constitute different manifestations of a more complex behavioural risk syndrome [12], which entails also social and contextual factors. For instance, family affluence appears to be related to adolescent sexual behaviours, although the extent and direction of that association is unclear [43]. On a related note, a very recent study shows that depression generally increases the risk of unintended pregnancy among young American women although this does not apply to those who are white or have a higher socioeconomic status [21]. From a behavioural risk syndrome perspective, it is reductive to abstract each relationship from the whole picture whereas it would be interesting to look into broader correlates of adolescent health and risk behaviours [12]. The disparities between adolescent males and females, and their cross-national variation, would be another interesting topic for further research. In fact, while boys are in general more likely than girls to report having had intercourse by the age of 15, that disparity is not observable in all countries or regions [43]. Besides, it would be interesting to study the relationship between psychopathology and sexual behaviours separately for girls and boys, as they cluster on different risk taking profiles [10].

Adolescent health practitioners need to be aware of the possibility that reproductive health risk is overrepresented among adolescents with psychopathology. This knowledge may be a useful tool for the early identification of young people who are exposed to specific risk or in need of tailored interventions [39]. Broad surveillance and comprehensive policies should also take into account the limitations of gateway effect interpretations and the diversity between boys and girls, who are particularly vulnerable because of the obvious peculiarities of female reproductive health outcomes. Possible interactions between key domains of European adolescents’ wellbeing have often been neglected [11, 12, 45]. However, clustering and correlates of their risk behaviours are cross-nationally consistent [40] and it is recognized that effective adolescent health strategies require intersectoral and multicomponent actions [37]. In this context, our findings highlight the need and potential for broad collaborative efforts to prevent disease and improve the current and future health of young people.

## Conclusions

Among European adolescent girls and boys, psychopathology is associated with early sexual initiation and reproductive health risk. This is particularly evident in case of more overt manifestations, such as self-injury and suicidality. Besides, the association between psychopathology and sexual initiation is particularly strong among pupils ≤15 years old and, for overt psychopathology, among pupils with low non-sexual risk behaviour profile or females. These findings should be taken into account when tailoring health-promoting or disease-preventing interventions for adolescents.
